# Neuralgia of the Trigeminus

**Published:** 1874-03

**Authors:** Louise Jacobi


### ARTICLE II.

### *Neuralgia of the Trigeminus.*

A Thesis presented to the Faculty of the Baltimore College of Dental Surgery, by Miss Louise Jacobi, for the degree of D. D. S.

I have selected the Neuralgia of the Trigeminus as the subject of my thesis, because with that nerve the dentist has most to deal, and should therefore, I consider, be well acquainted with all its normal and abnormal functions; and, further, because of a lack of a thorough knowledge of the nerve and its diseases, many teeth which could be saved for a long time, are sacrificed without affording the patient relief from suffering. I confess, in the beginning, that in many cases the characteristics of Neuralgia of the Trigeminus are not so clearly marked, that the dentist can always judge precisely, and often a very imperfect idea of them in some kinds of Neuralgia can be formed. But we are, however, able in the present advanced state of science to become so well acquainted with the subject, by proper reflection and careful examination, as to avoid many mistakes. We are greatly indebted for our knowledge of these subjects to the invaluable works of Charles Bell, Romberg, Valleix, Schuh, Wagner, Eulenberg, Benedict and others.

Having thus briefly stated my reasons for selecting the subject of my thesis, I now propose to speak directly of Neuralgia of the Trigeminus, and first to give the opinion, that it depends in every case upon a more or less delicate pathological change, (to which I will refer hereafter,) and when in the course of this thesis I speak of the symptoms of the disease, I will bear in mind that they are dependent upon this change.

The definition of Neuralgia could not up to the present time be given from an anatomical basis, and there was, therefore, a necessity to explain the disease from its symptoms; but as the symptoms do not show themselves in the same way in every case, the definition is varied by different authors. Bardeleben says: "Since the time of Chaussier the name of Neuralgia has been given to the hyperesthesia of the sensitive nerves, which shows itself in pain of great intensity in the course of a certain nerve." (*Bardeleben, Lehrbuch der Chirurgie und Operationslehre, II. Band, 6te Auflage, pag.* 302.) Bardeleben also says in his Aetiology, that the wounding of a nerve, pressure upon it from a tumor, or from any mechanical cause may result in Neuralgia. But according to Niemeyer, Neuralgia is a complication of symptoms, which does not always depend upon constant anatomical change. The definition given by him in his Aetiology is as follows: "When a blow, high or low temperature, or other injuries which strike the peripherial terminations of the nerves produce pain, or pain is produced by inflammation or diseases of the skin, the mucous membrane, or the parenchyma organs, we do not speak of it as

Neuralgia. But when we cannot take the irritations which have acted upon the peripherial terminations of the nerve as the cause of the pain, or it is probable that the irritating cause has acted upon the trunk itself, we call the pain then found in the extension of the nerve, Neuralgia." Mears, after speaking of some of the symptoms of Neuralgia, gives the followiug definition: "This painful condition existing in a nerve, is called Neuralgia. As it depends upon no constant anatomical characters, it cannot be defined as a disease. It is a combination of symptoms, and the causes which are engaged in its productions are various." (Trifacial Neuralgia, in the *Dental Times*, January, 1873, No. 3.) Other authors speak in the same way of the definition of the disease. It would require too much space and time to go further into this branch of my subject, and I therefore refer to the works of the acknowledged authors, and especially to *Romberg's Nervenkrankheiten, 3te Auflage, Berlin*, 1857, and *Valleix's Traite' des Neuralgies.*

The word Neuralgia comes from two Greek words, which mean nerve and pain; I adopt the meaning of the latter word, and call every pain in the nerve, no matter from what source it may arise, Neuralgia. Although I thus set for myself wide limits, I draw at the same time a distinction between several varieties or kinds. Every pain which is reflected in the nerve must not be confounded with Neuralgia, for the nerve then is only the physiological conductor, as in other forms of sensitiveness, and the conducting power, not the physiological part is afflicted, while in Neuralgia the nerve shares immediately in the affliction. But this sharing of the nerve in the pain could not always be proved by observations of anatomical change; as we up to the present time have derived our information of Neuralgia from the symptoms.

*Symptomatology.*—Neuralgia shows itself in most cases in somarked and peculiar a manner, that the practitioner can readily recognize it after having several times observed it; and yet, it does not remain so monotonous as might be at first inferred. Onthe contrary the pain manifests itself in many different ways; sometimes it is piercing, then again burning, itching, pricking, etc. It is strongly characteristic of the pain that the patient does not fix it at a certain point, but confesses its extension over a considerable district or region. The physician consequently finds the real aching point much smaller than the sufferer believes and states. Sometimes the pain radiates along the whole course of a nerve, according to the seat of the disease. Sometimes the pain radiates from one point of the nerve either to the periphery or to the centrum.

According to the direction of the pain Neuralgia has been divided into two kinds, ascending and descending. Valleix discovered first what he calls the points *douloureux* which show on pressure the most intense pain. But other authors have not always found these points, and they have been attributed to anatomical situation, of which I will speak under the head of anatomy.

Sometimes the localities of the pain are quite normal, but they are at other times pale, or redened, or swollen. The skin is at the point of pain a little stretched and dry, or relaxed and covered with a cold perspiration. Sometimes, also, the secretions of the mucous membrane and other glands which are connected with the afflicted nerve, increase, but the secretions seldom decrease. If the nerve is a compound one, the pain is often accompanied by convulsions. Often fibrilæ convulsions attend the disease like creeping sensations, as of the creeping of ants. On the other hand, there is also a feeling of relaxation, deafness, bluntness of teeth, observed with Neuralgia of the Trigeminus, conditions which arise from a depression of the sensibility. On account of the Neuralgia, there are in the region of the Trigeminus, exanthemes, as herper zoster developed; for instance, on the inferior maxillary, on the neck and in the clavicle part in which are found single branches of the fifth nerve.

The pain comes in paroxysms, which last only for a short time. They succeed each other sometimes very quickly and are very intense; they have an injurious influence upon the part in which the afflicted nerve is situated, so that it becomes often subject to atrophy or hypertrophy. This bad influence will by and by extend to the whole organism, and render persons of the most amiable tempers, morose and unbearable. Before an attack the patient may be either quite free from pain, or it may diminish in intensity so much that business can be resumed, from which the patient has perhaps been detained by the severity of the pain. The diseased parts become so stimulated, that their functions may be no longer normal; they may even become suspended. These paroxysms can be produced not only by the original cause, but by an accident, which may influence the diseased nerve. Trouble of mind is apt to produce them.

I have enumerated a sufficient number of the characteristic symptoms of Neuralgia. I will now endeavor to explain some details, keeping in mind, however, the one subject of my thesis.

*Aetiology*—Having considered the causes of Neuralgia, I desire to speak of the many conditions which will produce it. These conditions are so various, that they sometimes have no relation to each other, and yet they produce Neuralgia of the same form. The cause cannot always be determined; it is very probable that there is a delicate change of the fibrilæ of the axen cylinder, etc., which we are not able to observe with the naked eye, or even with the aid of the microscope. The same delicate changes may be supposed to exist in similar cases of nervous diseases. The real cause of Neuralgia is still wrapped in darkness, and on that account other causes are erroneously confused with it. After a while, Neuralgia seems to free itself, so to speak, from the originating causes, and to become independent of them, and often on this account the real cause remains undiscovered. Among the first ætiological causes of the bebeginning of Neuralgia, are the following:

Liability to Neuralgic attacks varies with the individuality of persons, but those predisposed to it are they who have an excess of nervousness, have anæmy, or are of scrofulous diathesis, or are very susceptible to changes of temperature. Most women between the ages of 30 and 50, are predisposed to Neuralgia. This fact cannot at present be explained. An explanation may be found in the greater nervous vital power, or in a subjective hysterical sensitiveness, which are oftener found in women than in men and children. The greatest attention to hysterical symptoms is necessary, as from them Neuralgic pain often originates in the form of toothache· We ought also to pay the greatest attention to rheumatic Neuralgia, with which we meet very often, because when it attacks the Trigeminus it produces the most violent toothache. Arthritical Neuralgia does not often exist; it belongs to rare cases. Other constitutional diseases, such as syphilis, may produce Neuralgia. For constitutional causeswe can add those coming from infections and poisonings, as with malaria. They are often followed by Neuralgia of the first branch of the Trigeminus. Neuralgia has also been observed in cases of typhoid fever, but not very often. It is difficult at the present time to explain how it happens that these fever diseases are followed by Neuralgia. But it is not so hard to explain the poisoning with lead, phosphorous, mercury, copper, etc., because the cauterization of the mucous membrane by those poisons may be the first ætiological cause.

The local causes of Neuralgia of the Trigeminus are to be found in the centre, in the course, or in the periphery of the nerve; they can be caused by pressure, by the thickening of the surrounding bones, exostosis on the bone or teeth, neuromen, cancers and other tumors, as hæmorrhagien, extravasats; also, inflammation of the parts in the neighborhood, inflammation of the sheath itself, irritation of the nerves by foreign bodies, twitching out by contracted scars, irregular and unequal distribution of blood, injuries, wounds, taking cold suddenly from a draught of air, long exposure to damp air, or the coldness from clothes often wetted by perspiration. Certain conditions in certain individuals are sometimes followed by acute Neuralgia, which comes and goes with the conditions themselves. To the causes already mentioned must be added those with which the dentist most often meets, namely, the caries of the teeth, and the chronic inflammation of the pulp or periost, hypertrophy of the cementum on the apex, which presses the nerve upon its entrance into the tooth, or thickening of the periost and mucous membrane of the antrum can produce Neuralgia in the superior anterior dental nerves, even when the teeth are sound, (Schuh.) Abnormal dentition, inflammation of the nerves of the gums during the cutting of the wisdom teeth, also the extraction of teeth, when not done in the most careful way can produce Neuralgia—(Wedl.) These are the mary causes which oftenest produce Neuralgia, and therapeutics are used with success as they are discovered.

*Anatomy.*—Of all nerves the Trigeminus is most subject to Neuralgia because of its anatomical situation. It is a compound nerve, consisting of two roots, different in their nature; the posterior, larger and sensitive one, originates in the *corpus restiforme*, and the *oliven* (*portio major;*) the anterior, smaller and motor one, originates in the *sinus rhom, boidalis*, (*portio minor.*) This nerve is after the formation of the *casserian ganglion*, which is much like the ganglion of the sensitive nerves of the spinal cords, chiefly formed by the sensitive root,* is divided into three branches, and on that account takes the name of Trigeminus or Trifacial nerve. They then go through bony canals. Neuralgia may be often attributed to this anatomical condition, and the least pressure upon the nerve will produce it. That this is a fact is proven by the successful excision of that part of the nerve exposed to pressure. This pressure upon the nerveby the bony canals can become active by the narrowing of the canals caused by the swelling of the bone, exostosis or inflammation of the lining membrane; or passive by the thickening of the nerve itself, or its excited sensibility.

*According to Shilling the sensitive fibres enter into the posterior columns, and the motor one into the anterior columns of the spinal cord.

Pressure upon the nerves may also be made in soft canals, as the fascias, through which the branches of the nerve pass, and by which they are sometimes more or less laced. The points *douloureux*, named by Valleix, are to be found in those canals, or to speak more accurately, in the foramens. In the Neuralgia of the three branches of the Trigeminus, it is very interesting to observe that these painful points are nearly always in the face in a line corresponding with the bony canals through which the branches of the nerve pass. The canals also effect the regular division of the blood, and so produce neuralgic pain. Until the blood gets from the veins of the spinal cord to the cava superior, it takes on the left side an indirect course, as it goes from the V. Hemiazigos into V. Azygos. The least resistance here causes the veins to over-fill, and Henle explains that this is the cause why intercostal Neuralgia is found oftener on the left than on the right side. From this predisposition of the left side, Henle draws the rational and ingenious conclusion, that the vene plexuses, which are in the neighborhood of the nerves, in passing through the above mentioned canals are over-filled, and thus exert an important influence upon the origin of Neuralgia. Henle also gives the opinion that the disease may be produced by the veins accompanying the nerves; he thereby explains the fact that the first branch of the Trigeminus is more subject to it than the second or third ones.

These are the anatomical conditions, which predispose certain nerves to the disease, and we may hope that in future we may gain more satisfactory explanations of the predisposition of individuals. Up to the present time the pathological anatomy has only given us in some cases such lesions as may be taken as causes. We have not yet been able to make a strict scientific division of Neuralgia either ætological or pathological anatomical. I do not think we are justified in dividing Neuralgia into two kinds, a pure or real one, when no cause can be stated, or unreal or impure when a cause can be found. We call Neuralgia descriptive or topographical-anatomical, and specify it after the diseases which it follows; sometimes also in reference to the causes which we are able to state. An irrational Neuralgia, if I may use such an expression, is that which has special reference to that in which we find anatomical lesions. Pathological anatomy can only show this lesion, but about idiopathological Neuralgia the anatomist has not been able to state anything. It is not well to believe that Neuralgia can exist in which the affected nerve retains its normal nature, and performs its normal functions. I think there must be an abnormity in its nature or in its chemical composition or vitality, which causes the disease. This abnormity can depend upon the different conditions of the nerve. The fibres may be atrophied or respectively hypertrophied, or the constituent parts may be in disproportion. We know from experiments that the least quantity of water first stimulates the nerve and then reduces it. Relaxation may follow excessive irritation of the nerve, and this may produce a thickening of the white substance of Swan, which presses then upon the axen cylinder and prevents it from performing its normal functions. A great many anatomical causes and explanations may be theoretically given, but they lack experimental proof and scientific basis, and up to the present time we look in vain to the experimental and cell pathology.

We are not able to determine if there be special pathological conditions of the ganglien of the Trigeminus which may be the cause of Neuralgia. But we know that they take a share in it, because we are able to determine the seat of the affection in certain cases by the functions of those nerve fibres which enter into them and which are experimentally fixed and explained by Magendie.

The alterations of the parts provided by the diseased nerve have been already refered to, and will be hereafter spoken of. I will only mention that in Neuralgia of the Trigeminus, the gums become spongy and bleed easily, that sometimes the teeth fall out; also it has a bad influence upon the eyes. As yet we have not obtained any certain observations of changes of the hair, which might be expected from the connection between the hair and teeth. Prof. Virchow spoke of such a connection in his speech about the wild men of Russia. *Berliner clinische Wochenschrift*, 21 *Juli*, 1873, *No.* 29. This connection is also found in cats; the fibres of the Trigeminus enter into the beard and render it very susceptible to the touch.

Some diseases, which may be followed by Neuralgia and have an anatomical pathological lesion, as inflammation, degeneration, tumor etc., have been spoken of, and I will now refer to only one anatomical condition, which according to Niemyer, even when only hypothetical, is very probable. He gives the opinion that rheumatic Neuralgia is based upon hyperæmy and an œdomentous swelling of the neurilem, which disappears after death. Benedict says in reference to the *tic douloureux*: We conclude from the isolated appearance of pain in the Trigeminus, and also from irradiating connections to the sensitive fibres of the muscles of the nerve facialis and the motor parts of the Quintus, that the *tic douloureux* is chiefly an affection of the Trigeminus nucleus and is also an intimate connection with the diseased condition of the sympathicus; and the results which are obtained by the compression and underbinding of the carotis refer generally to a chronic hyperæmy as the anatomical cause of the greatest probability.

*Diagnosis.*—It is by no means easy to make a diagnosis of the disease, because a plain but violent pain may falsely appear to be Neuralgia. We must therefore not be too ready to make a diagnosis of Neuralgia, if we have a patient who complains of pain in different places at the same time. The statement of the patient is only of value to the physician as it corresponds with practice and observations. A still greater care in a diagnosis is necessary, if we recognize a nervous disease with anatomical pathological condition which can be connected with Neuralgia and yet separated from it. I mean neuritis which has many special symptoms, but also many in common with Neuralgia. The characteristic symptoms are of great value in making a right diagnosis. It may therefore be here spoken of in comparison with those of Neuralgia. In neuritis we can establish always a positive lesion, which cannot always be done in Neuralgia. The course of neuritis is most acute,—that of Neuralgia most chronic. Neuritis does not as often end in paralysis as the latter. In acute neuritis the pain is without intermission, increases and does not change its character; in Neuralgia the pain is like a flash of lightning—now burning, then again piercing, etc. The neuritic pain will increase under pressure; the Neuralgic will not. The skin above the inflamed nerve is always red, burning hot and a little swollen. The nerve itself is sometimes a little swollen. But in the Neuralgia all these symptoms do not come so constantly to our observation. The result of Neuritis shows itself at the first beginning in the parts to which the inflamed nerve is distributed; then according as the nerve is a sensitive, motor or compound one, its conducting power is destroyed. In Neuralgia this can only occur after a long continuation of the disease. Along with Neuritis or after it, Neuralgia may develope and continue. As Neuritis can develope Neuralgia, so too Neuralgia can develope Neuritis. Neuritis may be, perhaps, an advanced step of a kind of Neuralgia, but they are under the present view of them, strictly separate. Neuralgia can also be complicated with other diseases, to which we must pay attention in determining the diagnosis. To refer more particularly to all possible complications would necessitate too great length.

To the symptoms of Neuralgia, already mentioned in Symptomatology, has been added in the Aethiology, the nature and original characteristics of the disease. We have now to determine the seat of affection, and this must be done after the observation of the symptoms. If the affection be a central one, that is, on the place where the nerve originates, all three branches of the Trigeminus are affected, but if the affected part is in the continuation of the nerve, one or two branches may be affected, and the other remain intact. We have not only to examine the sensibility and motor action, but at the same time to specially examine into a great many actions, which the Trigeminus, according to the experiments of Magendie, Budge, Valentine, Romberg and many other authors, performs during its course, and by finding an abnormity in them, we are able to determine the locality of the affection. This shows us that after thorough consideration of the complicated activities of the Trigeminus and other phenomena, we are able to make a diagnosis so accurate that one, not familiar with the whole nature of the nerve and the disease, would not be able to do. The more the affected point is peripheral the easier is the diagnosis, because the symptoms are then not so complicated as when its seat is central.

In Neuralgia of the first branch (*neuralgia frontalis*,) the patient complains of violent pain, beginning in the *fissura orbitalis* and radiating to the surrounding parts. Sometimes pustules and other exanthema are observed on the forehead. The upper eyelid is a little swollen, and during the attacks hangs down. The arteries beat, the eye is red, and the secretion of tears increases. Half of the nose is also affected.

In Neuralgia of the second branch (*neuralgia infraorbitalis*,) the pain comes suddenly and very violently from the *foramen infraorbitalis* to the under eyelid, the upper lip, the nose, and the teeth of the upper jaw, so that it is often mistaken for tooth-ache. During the attack, the skin is a little red and swollen; the pain is often so violent that it has a bad influence upon the whole organism, and this is increased by the disturbance of the functions of those parts supplied by the second branch, which are generally more actively employed than those parts supplied by the other branches. Convulsive movements of the muscles of the face are also observed, together with an increased secretion of saliva.

The Neuralgia of the third branch (*neuralgia maxillaris*,) affects mostly single branches of it, and rarely the whole branch. In former times the mistake was made of taking

Neuralgia of the Trigeminus for Neuralgia of the facial nerve, when in reality some branches of the maxillaris inferior were affected, which anastomose with the facial nerve behind the lower jaw. In the same way Neuralgia of the auricularis anterior was mistaken for facial neuralgic pain, and of course the cutting of the facial nerve did not give any relief. This method of cutting the seventh nerve can be traced to the times of Valpeaux, and even to a later date. We could not now make that mistake, because physiology has taught us that the facial nerve is purely a motor one. The mistake was made because the two nerves anastomose as before stated, and the pain irradiates from the Trigeminus to the parts supplied by the facial nerve. If the alveolaris inferior is affected, the pain locates itself mostly on the outer part of the lower jaw and chiefly around the foramen mentale, extends to the lower teeth and the whole half of the lower jaw. In the year 1872, I enjoyed the opportunity of observing a very interesting case in the clinic of the Pennsylvania College of Dental Surgery, in which the alveolar border of the left superior maxilla and the alveolus, and body of the left side of the inferior maxilla were affected, producing constantly recurring paroxysm of pain. The sufferer was an Englishman, 78½ years of age, who had suffered 38 years from Neuralgic pain. He had consulted physicians, but without relief. When he entered the clinic the Neuralgia was very intense, and he could not bear the least touch. The disease was produced by a thickening of the alveolar border. Prof. Mears removed the alveolar ridge and the operation gave the patient much relief. An extended account of the case is to be found in the report of Prof. Mears in the *Dental Times*, No. 3, 1873.

Comparing the Neuralgia of these three branches, I come to the following conclusion: Neuralgia of the first branch is most often met with in consequence of its anatomical nature; that of the third branch is the most infrequent; and Neuralgia of the second is the most intense, and therefore the most burdensome to the patient, causing excruciating pain.

Neuralgia *frontalis* gives the least trouble and pain to the patient.

It has been already said that Neuralgia is mostly of a chronic nature. The Aetiology has a great influence upon the course of the disease. Malaria Neuralgia is of an acute character. Except the paroxysm, Neuralgia is not equal in its entire course, remissions and exacerbations may be observed. The cause of this we do not know. Dry, bad and damp weather, and also the surroundings and humor of the patient, have a direct influence upon the remission and exacerbation. We know of malaria Neuralgia only as a regular type of attacks; but in other cases the paroxysms may be produced whenever an opportunity occurs. According to Niemeyer, the intervals between the attacks may be explained by the well known physiological fact, that a great excitation of the nerve exerts its excitability for some time, so that in Neuralgia the highest excitement changes with the exhaustion of the excitability of the nerve. In favor of this opinion of Niemeyer, is the fact, that after violent attacks the peripheral terminations of the nerves are for sometime to a certain extent without sensibility to external provocatives. It has also been observed, that after an attack has been produced by continual pressure upon a painful point, a repetition of it has not the same effect. Though this opinion of Niemeyer is an ingenious one, it does not fully explain it; this problem can only be theoretically explained, until the real cause of Neuralgic pain is known. This pain is connected with the abnormity in which the nerve is; it is then of no importance if the cause is the anatomical condition, the vital power or the chemical composition of the nerve. This disturbance in the phase of life in the nerve must have an external cause, and it leaves behind a predisposition to the attacks of the disease, even when a cure has been effected. This is the same as with catarrh of the stomach, intestines or bladder; if once there it will return upon the slightest provocation.

From what I have said it will be seen that the Neuralgia not being of a fatal nature, seldom terminates in a radical cure; of course, with the exception of malaria, rheumatic Neuralgia and cases of the same kind, which have an acute course. The prognosis accomodates itself to the ætiology. When the causes are known, and of that nature, that one can act upon them either surgically or therapeutically, the prognosis is easy; but in cases where the causes are not known, the prognosis cannot be determined. The general health of the patient is most important to the prognosis, and in some cases the greatest attention must be paid to the treatment for general health, because it is often the primary disease, and Neuralgia only the secondary. The prognosis depends then upon the nature of the constitutional disease, and is more favorable the younger the patient, and the more recent his illness. Even when there is no real danger of life, the disease is most burdensome and painful to the patient, making him low spirited, and giving him a great predisposition to other diseases, which may cause death.

*Therapeutics.*—I fear it would lead me too far, were I to dwell here more fully upon the therapeutics, and characterize the different remedies according to the effect they have in the present case; therefore I will speak only of the principles to be acted upon in the treatment of Neuralgia. The treatment of Neuralgia can be both medical and surgical, but must refer to the ætiology, and especially in Neuralgia of the Trigeminus; without such reference all remedies will be without result. The medical treatment must, if possible, act upon the primary causes; for only by detecting them can a radical cure be made. In cases of constitutional Neuralgia, which are produced by malaria, syphilis, hysteria, or other primaryca uses of that kind, specific remedies must be used; for Neuralgia from malaria, quinine; from syphilis, *kali jodatum*; from hysteria, preparations of valerian, etc These remedies do not forbid local treatment, but it must be used only as a help.

The indications which show themselves under treatment are numerous. First, all external irritation must be avoided.

This will be accomplished by covering the afflicted part with a proper substance, oil being the best. There should also be as much rest as possible, because the movement and use of the afflicted parts will often produce attacks. When the causes are known, the treatment must always be directed to a removal of them, but as they vary, the treatment must vary. If the cause is not known, then remedies such as experience teaches are proper, must be used, and the practitioner must accommodate the remedies as much as possible to the special cases. Attention must also be paid to the course of the disease; if it is acute, then those remedies which act, so to speak, acutely are to be applied,—they are vesicants. The application of hot and cold water, aconite, camphor, veratrin, etc., with short continued use are among the acute remedies. Bleeding must be resorted to with great precaution, and only after mature consideration. The treatment is much more difficult when the disease is chronic, because its place is then secured by long occupation, and the most radical remedies can only act very slowly, and sometimes without any result. In most chronic cases the benefits of watering places are often great, and the waters of Gastein, Rehme, Wildbad, Baden and other places, have a high reputation. Sulphur baths, and preparations of sulphur, are of great service in cases of Neuralgia from mercury, copper and lead poisoning, or from rheumatism—moor and salt baths and sea bathing are also of much service. They must be supported, however, by medical treatment, to result in benefit. The remedies used in chronic cases must be long continued; .they are arsenic, salicium, therebenthin preparations of zinc, *hyoseiamus* in pills. Carbonate of iron is also used with benefit in cases of anæmy and other weaknesses. Petroleum, ether, chloroform and opium are also beneficial. Among all these narcotics, morphine is of the greatest value, giving the patient speedy relief, by which it gains at once his confidence. Some use it as a secret remedy. A subcutaneous injection of morphine gives in all cases temporary relief, if not a permanent one. I recall here a case which was related to me. A lady, aged 36, had suffered for seven years from prosopalgy, and at last the pain increased so powerfully that her physician, who had exhausted many remedies without any beneficial result, took her to a Professor, whose name I do not feel authorized to give. The Professor agreed with the physician that the sufferings were caused by Neuralgia of the second branch of the Trigeminus, and recommended the subcutaneous injection of morphine. The first application gave much relief, and at the expiration of three weeks, a radical cure had been affected. This fact, communicated to me by a reliable person, shows that the subcutaneous injection of morphine can be used with great benefit, even when the causes are not known. Another important remedy remains to be mentioned, namely, electricity. This is also an aid in making a diagnosis, by reason of the reflex action of the muscles, which it produces, and the affected nerve can then be better located. The therapeutical value of electricity shows itself in a great many kinds of neuralgia. In some cases the inductive current acts; in some the constant current. How the electricity acts we do not yet know. I can not here enumerate all the probabilities which may be given for it; I refer, however, to the books upon the subject, and especially to the works of Benedict. When electricity is applied, the seat of the disease must before be accurately determined, then only by treating the *locus morbi* can we get a result. With much truth, Benedict says: "Unless the locality of the disease is known, the electhro-therapeutic is of very little value."—(*Benedict Electhrotherapie*, *Leipzig*, 1868, *pag*. 91.) Benedict treats Neuralgia of the Trigeminus in the following manner:—In Neuralgia of the second and third branches, he puts the copper pol behind the ear, and the zinc pol upon the *puncta dolorosa*. When the first branch is affected, the copper pol is generally placed upon the neck, and the zinc pol also upon the *puncta dolorosa*, or its radiations. In the majority of cases between 14 and 20, applications produce an effective cure, and sometimes sooner. If no relief is given at the beginning, then a continuance is not indicated.

Many reports of the curative power of electricity are to be found in the casuistic of the works of Benedict on electhro-therapeutic.

If one is sure, theoretically and practically, that therapeutics can give no relief, surgical treatment must be resorted to. This differs much in different cases according as the disease has itself originated. To present it in brief, the surgical operations may be divided into two groups; first, those in which the nerve itself is cut; and second, those in which the surrounding parts which press upon the nerve, are removed to relieve the nerve. I might here mention those cases in which only a surgical operation can give any hope of relief, but it would necessitate too great length, as I would be obliged to discuss the nature, the course and the characteristics of those cases.

In idiopathic Neuralgia, either Neurotomy or Neurectomy are used; the first consists in the cutting of the nerve, the other in the removal of a piece of the nerve. These operations are performed by different methods, and recommended by celebrated surgeons, as Langenbeck, (*Die subcutane Durchschneidung des nervus infraorbitalis in der fissura orbitalis inferior, Archiv. fur die clinische chirurgie, Band XI., pag.* 127.) Schuh, ( *Uber die Gesichts Neuralgien und uber die Erfolge der dagegen vorgenomenen Nerven resectionen, Wien,* 1858.) Linhardt, (*Compendium der Chirurgischen Operationslehre, Wien,* 1868.) Wagner, Vidal, Carnochan, Pancoast and others. Neurectomy is to be preferred in so much, that it gives a more certain satisfactory result, because when the nerve is only cut it easily heals. I refer again to the surgical works for information as to the manner of performing the different operations, and I close with the wish that the future may remove all that is problematical about Neuralgia of the Trigeminus, and take it out of the regions of symptomatology.

And in conclusion I return my most sincere thanks to the learned professors from whom I have had the honor to hear lectures. They may observe in this thesis the fruits of their patient and courteous instructions.

